# Guidelines for mitochondrial RNA analysis

**DOI:** 10.1016/j.omtn.2024.102262

**Published:** 2024-06-26

**Authors:** Amela Jusic, Zoi Erpapazoglou, Louise Torp Dalgaard, Päivi Lakkisto, David de Gonzalo-Calvo, Bettina Benczik, Bence Ágg, Péter Ferdinandy, Katarzyna Fiedorowicz, Blanche Schroen, Antigone Lazou, Yvan Devaux, on behalf of EU-CardioRNA COST Action CA17129, AtheroNET COST Action CA21153

**Affiliations:** 1 HAYA Therapeutics SA, Route De La Corniche 6, SuperLab Suisse - Batiment Serine, 1066 Epalinges, Switzerland; 2 Cardiovascular Research Unit, Department of Precision Health, Luxembourg Institute of Health, 1445 Strassen, Luxembourg; 3 Ιnstitute for Fundamental Biomedical Research, B.S.R.C. “Alexander Fleming”, Vari, 16672 Athens, Greece; 4 Department of Science and Environment, Roskilde University, 4000 Roskilde, Denmark; 5 Minerva Foundation Institute for Medical Research, 00290 Helsinki, Finland; 6 Department of Clinical Chemistry, University of Helsinki and Helsinki University Hospital, 00014 Helsinki, Finland; 7 Translational Research in Respiratory Medicine, University Hospital Arnau de Vilanova and Santa Maria, IRBLleida, 25198 Lleida, Spain; 8 CIBER of Respiratory Diseases (CIBERES), Institute of Health Carlos III, 28029 Madrid, Spain; 9 Cardiometabolic and HUN-REN-SU System Pharmacology Research Group, Center for Pharmacology and Drug Research & Development, Department of Pharmacology and Pharmacotherapy, Semmelweis University, 1089 Budapest, Hungary; 10 Pharmahungary Group, 6722 Szeged, Hungary; 11 NanoBioMedical Centre, Adam Mickiewicz University in Poznan, 61614 Poznan, Poland; 12 Department of Physiology, Cardiovascular Research Institute Maastricht, Maastricht University, ER 6229 Maastricht, the Netherlands; 13 School of Biology, Aristotle University of Thessaloniki, 54124 Thessaloniki, Greece

**Keywords:** MT: Non-coding RNAs, noncoding RNAs, mitochondria, gene expression, guidelines, microRNAs

## Abstract

Mitochondria are the energy-producing organelles of mammalian cells with critical involvement in metabolism and signaling. Studying their regulation in pathological conditions may lead to the discovery of novel drugs to treat, for instance, cardiovascular or neurological diseases, which affect high-energy-consuming cells such as cardiomyocytes, hepatocytes, or neurons. Mitochondria possess both protein-coding and noncoding RNAs, such as microRNAs, long noncoding RNAs, circular RNAs, and piwi-interacting RNAs, encoded by the mitochondria or the nuclear genome. Mitochondrial RNAs are involved in anterograde-retrograde communication between the nucleus and mitochondria and play an important role in physiological and pathological conditions. Despite accumulating evidence on the presence and biogenesis of mitochondrial RNAs, their study continues to pose significant challenges. Currently, there are no standardized protocols and guidelines to conduct deep functional characterization and expression profiling of mitochondrial RNAs. To overcome major obstacles in this emerging field, the EU-CardioRNA and AtheroNET COST Action networks summarize currently available techniques and emphasize critical points that may constitute sources of variability and explain discrepancies between published results. Standardized methods and adherence to guidelines to quantify and study mitochondrial RNAs in normal and disease states will improve research outputs, their reproducibility, and translation potential to clinical application.

## Graphical abstract

**Figure:**
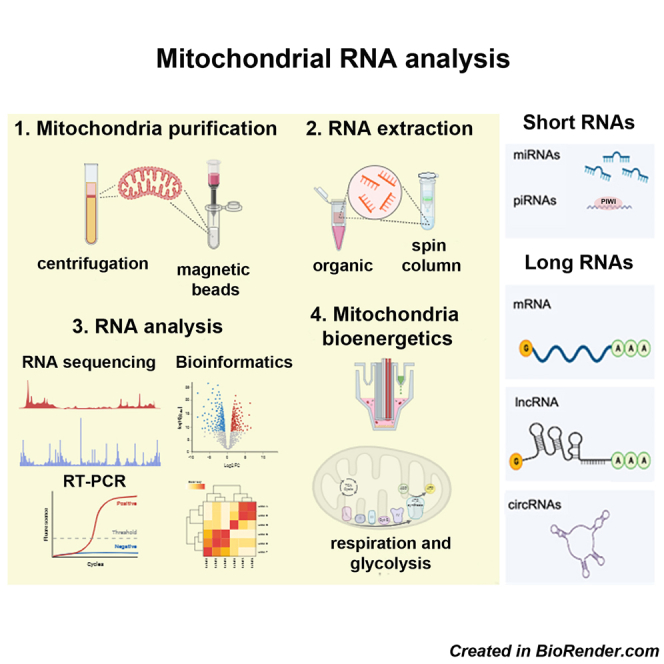

## Teaser

Devaux and colleagues discuss current pitfalls and challenges in the molecular profiling and functional characterization of mitochondrial noncoding RNAs. They summarize available techniques and provide guidelines for the establishment of standardized protocols for the study of these RNAs under normal and pathological conditions.

## Introduction

Mitochondria are cellular membrane-bound organelles originally derived from a proteobacterium during the evolution of the eukaryotic cell.^1^ They are responsible for the energy supply to the cells and are enriched in high-energy-demanding cells such as cardiomyocytes, hepatocytes, and neurons. Mitochondrial dysfunction is associated with cardiac, liver, and neurological diseases, as well as cancer, and is a potential source of novel therapeutic targets, which is currently under active investigation.

**Figure 1:**
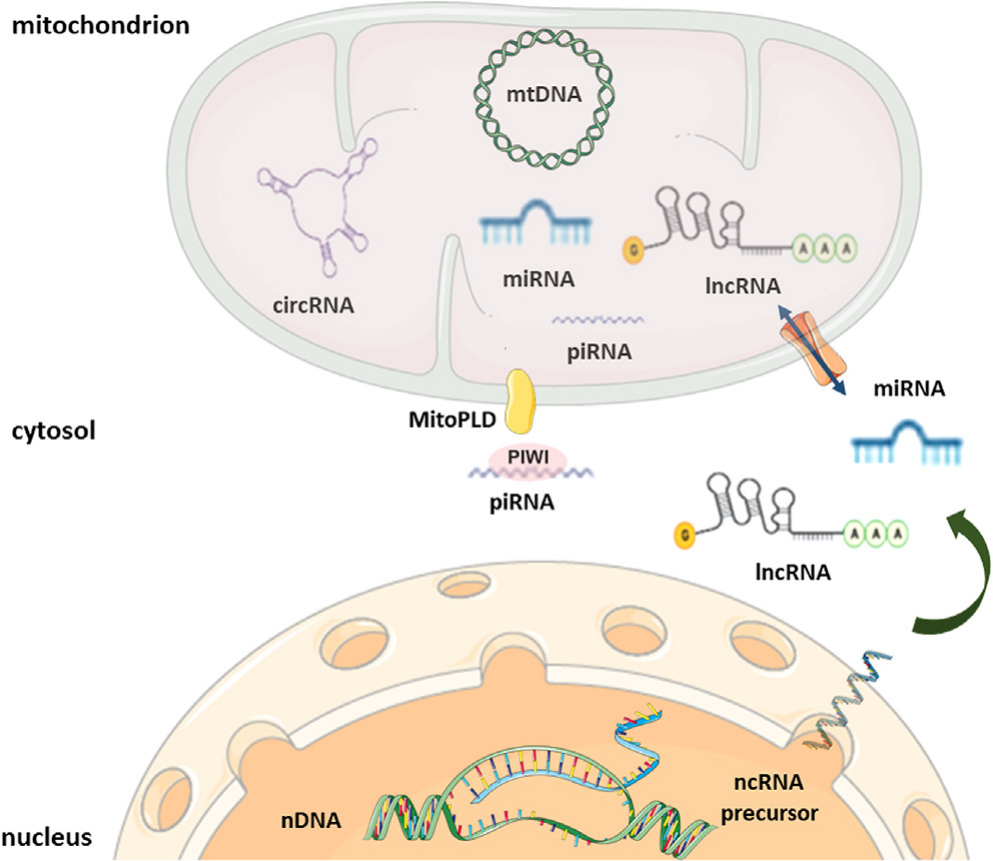
Biogenesis and targeting of mitochondria-related ncRNAs Mitochondria-related ncRNAs are of nuclear (nDNA) or mitochondrial DNA (mtDNA) origin. Nuclear genome-encoded lncRNAs and miRNAs are being processed in the cytosol and regulate mitochondrial physiology (anterograde signaling) by targeting transcripts/proteins outside or inside the organelle. The mechanism of import of ncRNAs in the mitochondrial matrix remains elusive. piRNAs encoded by the nuclear genome are being processed by MitoPLD at the surface of mitochondria. The mitochondrial genome encodes lncRNAs, miRNAs, piRNAs, and circRNAs that regulate mitochondrial and nuclear (retrograde signaling) gene expression. Whether mitochondria are autonomous for the maturation of these ncRNAs or they depend on cytosolic enzymes is a matter of debate (diagrams were adapted with permission from Servier Medical Art library, available under Creative Commons license).

Human mitochondria possess a circular genome, which is highly condensed and contains only 13 protein-coding genes, 2 transfer RNAs (tRNAs), and 2 ribosomal RNAs (rRNAs). Similar to nuclear DNA, mitochondrial DNA (mtDNA) is uniquely regulated by epigenetic factors such as DNA methylation and noncoding RNAs (ncRNAs).^2^^,^^3^^,^^4^^,^^5^^,^^6^^,^^7^ While ncRNAs lack protein-coding potential, they have extensive regulatory properties and act at multiple layers of gene expression.^8^^,^^9^ The improvement of RNA sequencing (RNA-seq) techniques has led to an increasing number of ncRNAs, including microRNAs (miRNAs), long noncoding RNAs (lncRNAs), circular RNAs (circRNAs), and piwi-interacting RNAs (piRNAs), being connected physically and/or functionally to the mitochondrial compartment (Figure 1).^10^^,^^11^

miRNAs are small ncRNAs with an average length of 22 nucleotides. They are transcribed as primary miRNAs and processed to precursor and mature miRNAs. In most cases, they mediate gene repression by interacting with the 3′ UTR of their pre-mRNA target and recruiting Argonaute (Ago) proteins to form the RNA-induced silencing complex (RISC).^12^^,^^13^^,^^14^ lncRNAs are noncoding transcripts that are longer than 200 nucleotides. They can be intergenic, intronic, antisense in protein-coding genes, or derived from pseudogenes. Many lncRNAs are spliced and polyadenylated. They participate in all aspects of genome organization, cell structure, and regulation of gene expression via interactions with other RNA molecules, DNA, and/or proteins.^9^^,^^15^^,^^16^ piRNAs are small ncRNAs of 24–31 nucleotides that interact with PIWI proteins, the germline subclass of Ago proteins. They mediate transposon silencing, preserve germline genome integrity, and participate in chromatin remodeling and mRNA degradation.^17^^,^^18^ circRNAs are single stranded, covalently closed RNA molecules, generated by the back-splicing of pre-mRNAs. Some circRNAs can be translated into peptides, whereas others act as transcriptional regulators, sponges for miRNAs, protein scaffolds, decoys, or recruiters.^19^^,^^20^ Table 1 summarizes key mitochondria-related ncRNAs and their roles in the regulation of mitochondrial genes and pathways. Further information on miRNAs targeting specific mitochondrial mRNAs can be found in Table S1 and the review paper by Jusic et al.^4^

**Table 1:** Summary of mitochondria-related ncRNAs and their role in mitochondrial physiology

ncRNA	Origin	Regulatory role in mitochondria	Reference
miR-30	nDNA	inhibits mitochondrial fission by suppressing p53 expression and its downstream target dynamin-related protein 1 (DRP1)	Li et al.^21^
miR-140	nDNA	promotes mitochondrial fission by suppressing mitofusin 1 (Mfn1) expression	Li et al.^22^
miR-125b	nDNA	reduces mitochondrial respiration and promotes mitochondrial elongation by silencing BCL2-interacting killer (BIK) and mitochondrial fission process protein 1 (MTP18), respectively	Duroux-Richard et al.^23^
miR-181c	nDNA	mediates respiratory complex IV remodeling by regulating gene expression of mitochondrial cyclooxygenase 1 and 2 (mt-COX1/2)	Das et al.^24^
miR-378	nDNA	downregulates the F0 component of ATP6	Jagannathan et al.^5^
miR-34a	nDNA	inhibits mitophagy by suppressing PTEN-induced putative kinase 1 (PINK1) expression	Tai et al.^25^
miR-27a and miR-27b	nDNA	inhibits mitophagy by silencing PINK1	Kim et al.^26^
miR-338	nDNA	decreases respiration of axonal mitochondria by targeting the 3′ UTR of cytochrome *c* oxidase IV (COXIV) and reducing its mRNA levels	Aschrafi et al.^27^
miR-15b	nDNA	prevents mitochondrial depolarization and mitochondrial ROS generation by silencing sirtuin 4 (SIRT4), and inducing its downstream targets cytochrome *c*, mitochondrial transcription factor 1 (TFAM), and nuclear respiratory factor 1 (NRF1)	Lang et al.^28^
let-7a	nDNA	destabilizes the mRNA of mitochondrial NADH dehydrogenase subunit 4 (ND4) and mediates metabolic reprogramming	Sharma et al.^29^
miR-1	nDNA	promotes the translation of cytochrome *c* oxidase subunit 1 (COX1) and mitochondrial NADH-ubiquinone oxido-reductase chain 1 (ND1) in differentiating myoblasts	Zhang et al.^30^
miR-21	nDNA	promotes the translation of cytochrome *b* (CYTB) in cardiomyocytes	Li et al.^31^
miR-5787	nDNA	promotes the translation of cytochrome *c* oxidase subunit 3 (COX3) and mediates metabolic reprogramming	Chen et al.^32^
miR-2392	nDNA	represses transcription of mtDNA and downregulates mitochondrial NADH-ubiquinone oxido-reductase chains 2, 4, and 5 (ND2-5), CYTB, and COX1	Fan et al.^33^
lncND5, lncND6 and lncCyt b	mtDNA	stabilizes their complementary ND5, NADH dehydrogenase subunit 6 (ND6), and CYTB mRNAs, respectively, by forming RNA-RNA duplexes	Jusic et al.,^4^^,^^11^^,^^34^ Ren et al.,^4^^,^^11^^,^^34^ Rackham et al.^4^^,^^11^^,^^34^
LIPCAR	mtDNA	regulates atrial fibrosis via TGF-β/Smad pathway	Wang et al.^35^
Kcnq1ot1	nDNA	reduces miR-378a levels and rescues ATP6 expression	Durr et al.^36^
Cerox1	nDNA	binds to miR-488-3p and promotes the expression and activity of mitochondrial respiratory complex I	Sirey et al.^37^
circRNA SCAR	mtDNA	binds to mitochondrial ATP synthase subunit β (ATP5B) and inhibits mitochondrial ROS production	Zhao et al.^38^
mecciND1 and mecciND5	mtDNA	mediates mitochondrial entry of proteins	Liu et al.^39^
mcPGK1	mtDNA	interacts with translocase of outer mitochondrial membrane 40 (TOMM40) and promotes mitochondrial import of phosphoglycerate kinase 1 (PGK1) to mediate metabolic shift from oxidative phosphorylation to glycolysis	Chen et al.^40^
circPUM1	nDNA	binds to ubiquinol-cytochrome *c* reductase core protein 2 (UQCRC2) and modulates mitochondrial respiratory complex III assembly	Gong et al.^41^

mtDNA, mitochondrial DNA; nDNA, nuclear DNA; LIPCAR, long intergenic noncoding RNA predicting cardiac remodeling; SCAR, steatohepatitis-associated circRNA ATP5B regulator.

Among the above-described ncRNAs, mitochondria-related miRNAs are the most studied and can be separated into three classes based on their genetic origin and subcellular localization: (1) nuclear-encoded miRNAs targeting mitochondria-related transcripts in the cytoplasm, (2) nuclear-encoded miRNAs translocating to mitochondria, and (3) mtDNA-encoded miRNAs.^4^^,^^42^ The two latter classes are also designated as mitochondrial miRNAs (mitomiRs). For instance, class 1 members, such as miR-30, miR-140, and miR-125b, regulate mitochondrial dynamics by targeting different components of the fusion/fission machinery.^21^^,^^22^^,^^23^ As an example of a class 2 miRNA, miR-181c was shown to translocate into the mitochondria of mouse cardiac myocytes and to regulate gene expression of mitochondrial cyclooxygenase 1 and 2.^24^ Kuthethur et al. identified 13 differentially expressed mitochondrial genome-encoded miRNAs (class 3) in breast cancer cell lines in comparison to a non-malignant breast epithelial cell line, and in breast cancer compared with normal tissue specimens.^43^ NcRNA-805, previously known as miR-805, was shown to be induced in response to cellular stress and to play a role in the regulation of the tricarboxylic acid cycle.^44^ In response to type 1 diabetes-induced hyperglycemia, mitomiR-378 regulates the mitochondrial-encoded F0 component of ATP6 in cardiomyocyte HL-1 cells.^5^ Multiple studies suggest an association of mitomiRs with various diseases including cancer and cardiovascular diseases indicating their clinical importance as biomarkers or therapeutic targets.^4^^,^^45^

In addition to miRNAs, several lncRNAs have been related to mitochondria.^6^^,^^46^^,^^47^^,^^48^ lncRNAs potentially encoded by the mitochondrial genome have been identified in publicly available transcriptome databases or detected by northern blot and reverse-transcription quantitative PCR (RT-qPCR).^34^^,^^49^ Novel lncRNAs have been associated with cardiovascular and liver diseases. For instance, long intergenic noncoding RNA predicting cardiac remodeling has been proposed as a biomarker for patients with heart failure.^50^^,^^51^ Liu et al. reported that mtDNA-encoded circRNAs serve as molecular chaperones for the folding of proteins imported into mitochondria, and mediate mitochondria-to-nucleus communication.^39^ Mitochondria-localized circRNA steatohepatitis-associated circRNA ATP5B regulator binds to ATP synthase subunit β and inhibits mitochondrial reactive oxygen species (ROS) production and activation of liver fibroblasts, a critical step in the progression of non-alcoholic steatohepatitis.^38^^,^^52^ Latest next-generation sequencing (NGS) analyses leveraged piRNAs generated from mitochondrial tRNA genes in mouse primordial germ cells and somatic cells, as well as in human normal and cancer cell lines.^53^^,^^54^ It was suggested that these mtDNA-encoded piRNAs play a role in anterograde and retrograde signaling between mitochondria and the nucleus. Of note, mitochondria have been described as essential to piRNAs biogenesis, which involves the nuclease activity of MitoPLD/Zucchini, a mitochondria-anchored member of the phospholipase D superfamily.^55^^,^^56^^,^^57^

Despite accumulating evidence on the existence of mitochondrial ncRNAs, there are significant conceptual and technical challenges in studying their biological impact under physiological and pathological conditions. Our knowledge of the biogenesis and processing of these RNAs, and their transport in and out of mitochondria, is limited by important gaps and numerous controversies (Figure 1).^10^^,^^58^ To date, there is no proof of the presence of enzymatic activities involved in the processing and maturation of ncRNAs inside the mitochondrial matrix. Although components of the RISC including Ago2 have been shown to localize in mitochondria, there is no conclusive evidence of miRNA-mediated regulation of mitochondrial transcripts taking place in the mitochondria matrix or in the cytoplasm.^30^^,^^59^^,^^60^ Different facilitators of RNA import into the mitochondrial compartment have been suggested, including the ribonuclease polynucleotide phosphorylase and the RNA binding protein GRSF1, but how ncRNAs are being shuttled between mitochondria and the cytoplasm or the nucleus remains elusive.^61^^,^^62^ Last, but not least, it is well established that miRNA-mediated repression of gene expression is relatively mild.^63^^,^^64^ Many newly identified mitochondrial ncRNAs show low abundance, which renders their functional relevance questionable, at least in the specific biological context (cell type, growth conditions) they are being studied.^65^^,^^66^

Currently, there are no standardized protocols and guidelines to conduct expression profiling of mitochondrial RNAs and deep functional characterization. The main obstacles to studying mitochondrial RNAs are (1) a lack of techniques allowing isolation of uncontaminated mitochondria/mitoplasts from other membrane-bound vesicles, especially from small amounts of tissue, (2) mitochondrial ribonuclease activities degrading mitochondrial RNAs during the isolation process, (3) a lack of standardized assays for RNA-seq and RT-qPCR, and (4) a lack of reliable reference genes for relative expression quantification. These technical challenges hinder the accurate profiling of RNAs (especially ncRNAs) in the mitochondrion, which explains the ongoing debate and controversies on the existence of different types and functions of RNAs in this organelle.

**Figure 2:**
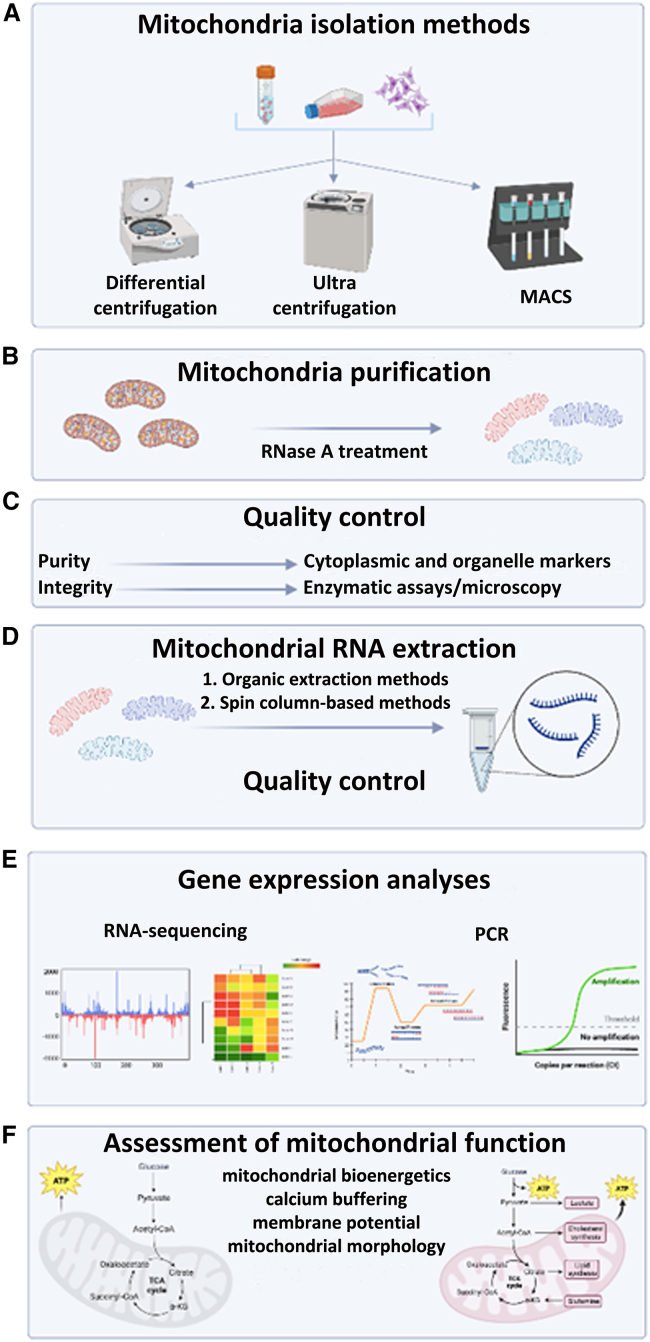
Workflow of mitochondrial RNA expression profiling (A) Isolation of mitochondria by differential centrifugation, ultra-centrifugation using density gradients, or antibody-mediated capture using magnetic separation (MACS). (B) Mitochondria purification after RNase A treatment to remove cytosolic RNAs from the outer mitochondrial membrane. (C) Quality control of mitochondrial preparations using enzymatic assays, microscopy, and enrichment analysis of mitochondrial markers. (D) Mitochondrial RNA extraction and quality control. (E) Gene expression analyses including RNA sequencing and RT-qPCR. (F) Assessment of mitochondrial function (created with BioRender.com).

To address these challenges, experts from the EU-CardioRNA and AtheroNET COST Actions networks summarize current techniques with an emphasis on critical steps during the experimental procedure that are prone to introduce bias and/or artifacts in the study of mitochondrial ncRNAs.^67^ Figure 2 displays the workflow of mitochondrial RNA expression profiling, comprising five steps that constitute the first technical sections of this article, starting from (1) mitochondria isolation to (2) mitochondrial purification, (3) quality control, (4) RNA extraction and quality control, and (5) RNA expression profiling. The last section includes recommendations on the assessment of the role of RNAs in the regulation of mitochondrial function as well as recent advances in detecting and targeting these ncRNAs inside the mitochondrial compartment (see functional analyses, Figure 2F).

### Mitochondria isolation methods

The starting and critical point in mitochondrial RNA expression profiling and functional characterization is the isolation of highly pure mitochondria (Figure 2A). Different techniques have been developed to extract mitochondria from cells and tissues with most of them relying on differential centrifugation (DC), ultra-centrifugation with density gradients, affinity purification of the organelle by magnetic sorting, or free-flow electrophoresis (FFE).

#### DC

DC is a widely employed technique for the isolation and purification of mitochondria for functional analyses such as the measurement of O_2_ consumption, transmembrane potential, ROS formation, ATP production, and swelling.^68^ The DC method is based on a gentle homogenization of the sample followed by a series of centrifugations of increasing centrifugal force to pellet crude mitochondria. Several DC protocols for mitochondria isolation have been described previously.^68^^,^^69^^,^^70^^,^^71^^,^^72^

An important factor to consider during mitochondria isolation for RNA expression profiling studies using DC is the mechanical force applied for tissue or cell homogenization, which varies by cell and tissue type. For example, soft tissues such as the kidney, brain, and liver require gentle mechanical forces, while harder tissues such as cardiac and skeletal muscles require much stronger mechanical forces.^73^ Of note, all buffers used for homogenization and centrifugation should be ice-cold and have a physiologically relevant pH with an ionic and osmotic strength compatible with the cytosol. Fernández-Vizarra et al. described a detailed protocol for mitochondria isolation for biogenetic studies from brain, heart, kidney, liver, and cultured cells.^73^ The advantage of DC is that the technique is inexpensive and relatively quick (1–2 h) compared with other techniques. However, this technique has some limitations: (1) it requires a large amount of sample, restricting its use for tissue biopsies or primary cells and (2) mitochondria can be contaminated with other cellular components which require additional washing and centrifugation steps.

Several commercially available mitochondria isolation kits exist that are based on the DC approach. These kits require standard laboratory equipment and constitute theoretically quick and simple procedures, yielding pure and functional mitochondria from cell culture and tissue samples that can be further used in almost any downstream application. Kits are attractive when limited starting material is available and a large number of samples must be processed in a reproducible manner in terms of mitochondrial quantity and quality. Mitochondrial integrity is well preserved, but the yield may be lower compared with manual procedures. Although no special equipment is required, the relatively high cost of kits can be a limiting factor for their systematic use.^70^^,^^74^

#### Ultra-centrifugation with density gradients

Density gradient centrifugation (DGC) relies on the combination of basic mitochondrial extraction using DC with further purification employing one or more gradients.^75^^,^^76^ A detailed protocol on mitochondrial isolation using DGC has been described by Graham.^77^ In DGC, the tissue or cellular extract is layered over a sucrose or a Percoll cushion and centrifuged at a certain speed (17–31,000 × *g*) causing the mitochondria to be isolated from other cellular components according to their densities. This method is often used to isolate brain mitochondria with very low contamination from synaptosomes. Thus, while the purity of mitochondria isolated by DGC is higher than for DC and can meet criteria for some applications, such as mitochondrial proteomics analyses, investigation of mitochondrial morphology and mitochondrial apoptosis, a limitation of DGC is the lower yield of purified mitochondria compared with DC.^78^ Contamination by other subcellular particles may necessitate additional washing and centrifugation steps (see quality control of mitochondrial preparations). In addition, the DGC method is more laborious and time-consuming than DC.^72^

#### Mitochondria isolation based on magnetic-activated cell sorting

Mitochondria isolation based on magnetic-activated cell sorting (MACS) is a separation technique commonly used for isolating different types of cells or organelles based on binding to antibodies coupled to paramagnetic beads. The MACS technique ensures the isolation of mitochondria of good purity. It uses magnetic beads coated with antibodies targeting the translocase of the outer mitochondrial membrane protein 22 (TOMM22) at the surface of mitochondria.^79^ When optimized, this procedure can be completed in less than 30–45 min with a success rate, purity, and integrity significantly higher compared with DGC for neuronal synaptosomal mitochondria although not for total brain mitochondria.^80^ In addition, the MACS method allows the extraction of mitochondria from tissues and cells with low mitochondrial content.

Among the current mitochondrial extraction and purification methods, the magnetic bead method shows better performance in impurities elimination including microsomes and peroxisomes compared with the DC method.^81^ Reports also suggest that isolated mitochondria by MACS protocols are well suited for mitochondrial RNA expression profiling studies.^59^^,^^82^ MACS can be automated and standardized. However, the MACS method requires substantial amounts of antibodies, which can lead to prohibitive costs for studies including numerous samples. Also, the quantity of tissue or cells that can be used as input material is limited, thereby reducing the yield of isolated mitochondria.

#### FFE

FFE is a well-established method that allows the isolation of mitochondria with high resolution and purity compared with other methods.^83^^,^^84^ In FFE, a sample is continuously fed into a chamber filled with a flowing buffer. An electric field is applied perpendicular to the flow, causing charged particles to separate into distinct streams based on their mobility. However, commercial FFE systems typically require large sample volumes, which can be restrictive when only small sample sizes are available, such as rare cells or small tissue samples.^85^ In addition, extended exposure to electric fields and chemicals could potentially compromise the structural integrity of mitochondria due to their delicate nature.^85^

Advantages and limitations of mitochondria isolation methods are summarized in Table 2.

**Table 2:** Advantages and limitations of mitochondria isolation methods

Mitochondria isolation		
Method	Advantages	Limitations
differential centrifugation	✓cost-effective ✓quick	✕ a large amount of sample required ✕ high contamination by other organelles
ultracentrifugation with density gradients	✓high mitochondria purity	✕ time-consuming ✕ laborious ✕ low yield of mitochondria
affinity purification by magnetic sorting	✓high reproducibility ✓quick and effective	✕ limited sample quantity ✕ expensive at large scale
free-flow electrophoresis	✓high purity and reproducibility	✕ prolonged exposure to electromagnetic field can damage mitochondrial integrity ✕ requires high sample input

### Mitochondria purification

Successful mitochondrial RNA expression profiling depends on the preparation of highly purified mitochondria with low amounts of contaminating cytosol and organelles, as other organelles also contain RNAs. Additional purification procedures (Figure 2B) to remove outer mitochondrial membrane-associated RNAs using digestion with RNase A, followed by incubation with sodium dodecyl sulfate (SDS) and/or proteinase K to inactivate exogenous and resident RNases are required.

Detailed protocols to eliminate cytosolic RNAs from the outer mitochondrial membrane using RNase A treatment described previously.^59^^,^^86^^,^^87^ For downstream mitochondrial RNA isolation, RNase A must be removed to prevent later degradation of the purified intra-mitochondrial RNA. This can be achieved by proteinase K treatment and requires subsequent washing twice in suspension buffer followed by recovery by centrifugation (e.g., 13,000 × *g* for 2 min at 4°C) to remove the protease. While proteinase K and SDS combined with elevated temperatures (100°C) can be used in protocols for mitochondria preparation for RNA extraction, these conditions impact RNA stability. To overcome this obstacle, Huang and Wang proposed a method using SDS lysis coupled with a milder temperature (70°C) incubation, which denatures most proteins without causing RNA instability.^88^ A subsequent proteinase K digestion degrades surface proteins, providing a reliable protocol to isolate mammalian mitochondrial RNA suitable for RT-qPCR and other downstream assays. For each experimental procedure, it is important to assess the potential consequences of incomplete RNase A inactivation on mitochondrial RNA expression profiling.

### Quality control of mitochondrial preparations

Quality control of mitochondrial preparations constitutes an obligatory step in any pipeline investigating mitochondrial RNA expression. The quality control of mitochondrial preparations (Figure 2C) includes evaluation of purity, defined as relative enrichment in mitochondrial markers and by the absence of contaminating proteins from other organelles such as lysosomes, peroxisomes, endoplasmic reticulum, and endosomes. Possible contaminants of mitochondrial preparations by other organelles can be assessed by measuring the activity of acid phosphatase (lysosomes), glucose-6-phosphatase (microsomes), or catalase (peroxisomes).^89^

Mitochondrial integrity is defined by the measurement of functional characteristics and may be monitored by measuring activity of the mitochondrial matrix enzyme citrate synthase and the inner membrane protein cytochrome *c* oxidase.^59^ Integrity of mitochondrial membrane structure may also be assessed by confocal microscopy following immunostaining against mitochondrial markers, but also transmission electron microscopy or atomic force microscopy to detect ultrastructural abnormalities, such as disrupted cristae and mitochondrial swelling.^90^^,^^91^^,^^92^ However, many published studies investigating mitochondrial RNA expression did not include these quality control steps systematically. Since sequencing techniques are very sensitive, they can easily detect small amounts of contaminating cytosolic RNAs leading to possible data misinterpretation. Thus, obtaining pure mitochondrial preparations and an RNA pool devoid of cytosolic RNAs is a prerequisite for reliable RNA studies.

Commonly used methods for monitoring the purity of mitochondria regarding RNA and protein content are RT-PCR and western blot analysis.^90^ In experimental RT-PCR settings, RNA isolated from cytoplasmic and pure mitochondria fractions are analyzed for the presence of, e.g., glyceraldehyde 3-phosphate dehydrogenase (unambiguous cytoplasmic marker) and mitochondrial genome-encoded markers (e.g., 16S rRNA, MT-CYB). Similar markers can be used for western blotting.

### RNA extraction from mitochondria and quality control

#### RNA extraction methods

Numerous protocols have been proposed to isolate RNA from mitochondria based on organic extraction, spin columns, or paramagnetic bead-based extraction methods (Figure 2D).^93^ Geiger and Dalgaard described the isolation of mitochondrial RNAs from rat liver tissue and HepG2 cells based on phenol-guanidinium isothiocyanate-chloroform RNA extraction.^87^ An important factor to consider for the selection of appropriate procedures for mitochondrial RNA isolation is the low input of mitochondria for RNA extraction. Although mitochondria are highly abundant in brain, heart, or liver tissues, pre-processing preparation steps and RNase A treatment of mitochondria before RNA extraction result in significant loss of material, which consequently may lead to low yield of mitochondrial RNA. In line with that, several phenol-free commercial kits allowing RNA extraction from low input of mitochondrial samples may be recommended (Table 3).

**Table 3:** Commercially available automated and manual extraction kits for the isolation of total RNA including small RNAs from low-input cell and tissue samples

RNA extraction kit	Manufacturer	Input	Method	RNA biotypes
QIAzol TRI Reagent TRIzol	QIAGEN Sigma-Aldrich Thermo Fisher Scientific	up to 100 mg per 1 mL reagent or 1 mL per 10^7^ cells	manual	total RNA including miRNAs
RNeasy Plus Micro and Mini Kits	QIAGEN	5 × 10^5^ cells or 5 mg (Micro kit) 10^7^ cells or 30 mg tissue (mini kit)	manual and automated	total RNA including miRNAs
RNAqueous Total RNA and Micro Isolation Kit	Thermo Fisher Scientific	1–75 mg of tissue or from 10^2^–10^7^ cells	manual	total RNA including miRNAs
Arcturus PicoPure RNA Isolation Kit	Thermo Fisher Scientific	laser-capture micro-dissected samples and larger samples	manual	total RNA
MagMax mirVana Kit	Thermo Fisher Scientific	various low-input samples	manual and automated	total RNA including miRNAs
Total RNA Purification Plus Kit	Norgen	various low-input samples	manual	total RNA including miRNAs

Organic extraction methods, such as the phenol-guanidinium isothiocyanate-chloroform method, offer superior RNA yields and stable isolation suitable for downstream applications. However, these protocols are susceptible to RNA contamination with phenol and other contaminants, and they are generally time-consuming compared with spin-column-based methods. On the other hand, spin-column-based methods provide reduced contamination risk due to the enclosed system but may provide lower RNA amounts compared with organic extraction.^94^^,^^95^ Commercial kits designed for mitochondrial RNA isolation enable RNA extraction from low-input mitochondrial samples. However, they tend to be more expensive than manual extraction methods, and there is variability in performance among different kits.

Overall, the choice of RNA isolation method should consider factors such as the RNA species of interest (e.g., short or long RNAs), downstream applications (e.g., NGS or PCR), and considerations of time, cost, and sample input. Each method has its advantages and limitations, and the selection of the most appropriate method for specific study requirements should be carefully considered.

#### Quality control of mitochondrial RNA

Fast, accurate, sensitive, and specific quantification of integrity, quality, and purity of mitochondrial RNAs for downstream gene expression analyses can be achieved using Qubit Fluorometers (high-sensitivity and/or microRNA kits) and automated electrophoresis methods (Agilent Bioanalyzers or TapeStation).^96^^,^^97^ Due to low RNA yields from mitochondria, standard optical density methods (OD_260nm_) may not be applicable for accurate quality control. RNA integrity is critical for subsequent profiling, and ongoing degradation in low quality samples often leads to overestimation of RNA concentration.^98^

### Mitochondrial RNA expression analyses

Molecular profiling methods of mitochondrial RNAs include NGS, PCR-based methods, and microarrays (Figure 2E). NGS is a time-consuming approach, requiring a great investment in subsequent bioinformatic analyses, but can generate information on novel miRNAs, and detect different isoforms (isomiRs) and post-transcriptional modifications. PCR-based analyses and microarrays on the other hand are compatible with quantification of known RNAs. A summary of the advantages and limitations of these approaches is presented in Table 4.

**Table 4:** Advantages and limitations of different approaches for mitochondrial RNA expression analysis

RNA expression analysis		
Method	Advantages	Limitations
next-generation sequencing	✓high throughput ✓identification of novel ncRNAs and their modifications	✕ laborious and long bioinformatic analysis ✕ bias introduced during library preparation
single-molecule real-time sequencing/Nanopore sequencing	✓generation of full-length transcripts ✓identification of novel ncRNAs and their modifications ✓amplification bias bypassed	✕ laborious and long bioinformatic analysis ✕ high cost
qPCR	✓rapid ✓cost-effective ✓absolute (standard curve required) and relative quantification	✕ limited to known ncRNAs ✕ lack of reliable reference RNAs ✕ sensitive to RNA quality
droplet digital PCR	✓no standard curve required for absolute quantification ✓discriminatory power for low-input or low-quality samples	✕ limited to known ncRNAs ✕ low dynamic range compared with qPCR ✕ high cost

#### NGS

Small RNA-seq has become a popular method for high-throughput miRNA profiling.^99^^,^^100^ Nevertheless, NGS data on mitochondrial ncRNAs often fail to be reproduced, and comparison of datasets obtained by different library preparation approaches requires great caution.^101^^,^^102^^,^^103^^,^^104^^,^^105^ Efficient representation of miRNAs in the library to be sequenced depends on the RNA isolation method (the use of spin columns capable of retaining RNA molecules that are greater than 10 nucleotides is highly recommended), rRNA depletion and enzymatic ligation of adapters to the RNA molecules.^106^^,^^107^^,^^108^ RNA ligases show differential preference for miRNAs based on their structure, sequence, strand orientation, and post-transcriptional modifications, leading to adapter ligation bias, and subsequent reverse transcription and amplification biases.^109^^,^^110^ Thus, some miRNA species or variants of a given miRNA may be favored during library preparation at the expense of others, compromising their quantitative analysis. The use of randomized adapters and/or the addition of polyethylene glycol in the ligation reaction allow overcoming the ligation bias.^101^^,^^102^^,^^103^ The sensitivity of commercially available library preparation kits has been greatly improved to overcome the limitations of low-input mitochondrial RNA (input <100 ng of total RNA or sub-ng amounts of miRNA). However, none of these kits is able to accurately reflect the relative amounts of all miRNAs in the original sample.^103^ Increasing sequencing depth improves sensitivity, but also favors the detection of transcriptional noise. Indeed, a substantial number of novel miRNAs identified by RNA-seq fail to be confirmed in subsequent studies.^101^^,^^102^ Experimental validation of newly identified miRNAs is required prior to any further functional study and involves the detection of both the precursor and mature forms of these RNAs (at endogenous levels or after ectopic expression) by northern blot.

Third-generation, long-read sequencing approaches, such as single-molecule, real-time sequencing (available from Pacific Biosciences), and Nanopore Sequencing (available from Oxford Nanopore Technologies), provide full-length transcripts, bypassing the amplification bias and the need of assembly.^111^^,^^112^^,^^113^ Their use in the study of mitochondrial ncRNAs is so far very limited due to their high cost, but they hold great promise for the identification of novel RNA molecules/isoforms and RNA modifications.^49^^,^^114^

#### PCR-based methods

RT-qPCR is considered a routine technique to measure gene expression in various sample types including mitochondria. Although RT-qPCR is widely used, several factors may lead to quantification bias including: (1) variations in protocols, reagents, sample quality, and instruments, (2) inconsistent data analysis, (3) the investigation of samples containing low amounts of the template with small expression differences of 2-fold or less, (4) sample quality heterogeneity, which affects the efficiency of RT-qPCR, and (5) differences in interpretation or data-analytical protocols within and across laboratories.^115^^,^^116^

These limitations also apply to RNA-seq and some of them may be overcome using droplet digital PCR (ddPCR), a method that provides ultrasensitive nucleic acid detection and absolute quantification. Although both techniques, RT-qPCR and ddPCR, utilize Taq polymerase in a standard PCR reaction and pre-validated primer or primer/probe assays, they have major differences: (1) in ddPCR, samples are portioned into thousands of individual droplets that undergo PCR amplification and (2) following PCR, each droplet is analyzed using Poisson statistic to determine the target gene concentration in the tested sample.^115^ ddPCR allows direct and independent quantification of target genes without standard curves and resolves quantification of low abundance targets, which are below the detection limits of other PCR platforms. Thereby, an advantage of ddPCR technology is its discriminatory power for low-target quantitation (quantification cycle [Cq] ≥ 29) including mitochondrial RNAs as well as low-input samples and/or samples containing variable amounts of chemical and protein contaminants.^117^ A low dynamic range as compared with other PCR methods can be a limitation of ddPCR, and its use at a routine level is restrained by the elevated costs for instrumentation and maintenance.

#### Gene expression analysis

The two most widely used methods to analyze RT-qPCR data are absolute quantification and relative quantification. While absolute quantification determines the input copy number, usually by relating the PCR signal to a standard curve, relative quantification relates the PCR signal of the target transcript to that of another transcript used as a control. This process is known as normalization and is a crucial step in RT-qPCR analysis.

The most common method to normalize RNA expression data is to use stably expressed reference genes as internal controls for monitoring RNA extraction, reverse transcription, and qPCR efficiency.^118^ It is recommended to use multiple reference genes instead of a single one to obtain more accurate results.^118^^,^^119^ However, to date, no universal reference genes and no common rules for PCR normalization have been defined, especially for mitochondrial RNAs, and even less for miRNAs and other ncRNAs. This gap limits the comparison of results between studies and is still challenging to address. Different normalization strategies may give different results and even lead to misinterpretation of data.^120^^,^^121^ Reference genes that are stable under some conditions may change significantly in other conditions or diseases. Thus, validation of the optimal reference genes in each individual experimental and clinical setting is important to limit biases.^118^^,^^122^ Strikingly, only a few studies justify the choice of reference gene(s) and report its/their stability in their experimental setting. Different algorithms, including Normfinder, geNorm, and BestKeeper, can also be used for the selection of optimal normalization genes for specific experimental conditions.^119^^,^^123^^,^^124^ In a comparative study, these algorithms converged to the same most stable and less stable reference genes, but the ranking was different depending on correction against PCR efficiency.^125^

As far as mitochondrial RNAs and particularly mitomiRs are concerned, the choice of optimal reference genes depends on whether the mitomiR expression is studied in mitochondrial, whole-cell, tissue extracts or plasma/serum. U6 and other small nucleolar RNAs (snRNAs) are commonly used for the normalization of miRNA expression in cells and tissues, and they have been used for the normalization of mitomiRs expression as well.^5^^,^^29^^,^^33^^,^^43^^,^^126^^,^^127^^,^^128^^,^^129^ The use of nucleus-enriched snRNAs as normalizers for RNA species purified from mitochondrial extracts is inappropriate, because they are substantially, yet variably, depleted in mitochondrial extracts. Using TaqMan miRNA RT-qPCR arrays and the global mean normalization method, Wang et al. observed that U6 snRNA levels were 13-fold lower in mitochondria compared with the cytosolic fraction of hippocampal tissue.^130^^,^^131^ Moreover, the expression of snRNAs may not be stable in all pathological conditions and their use as reference genes may introduce bias in miRNA expression analysis.^121^^,^^122^ Furthermore, snRNAs have different biochemical properties compared with miRNAs, which may lead to different efficiencies in RNA extraction, RT reaction, and qPCR. 5S and 12S rRNAs are also commonly used as reference genes for miRNA quantification.^32^^,^^43^^,^^132^ 12S rRNA is a mitochondrial gene product and 5S rRNA is present in both cytosolic and mitochondrial fractions. Das et al. used 12S rRNA as a reference gene when comparing miR-181c expression in mitochondrial compared with total heart tissue fraction and 5S rRNA when comparing miR-181c expression in cytosolic and mitochondrial fractions.^132^ A similar normalization strategy has been used in cancer cell lines.^32^

Stably expressed miRNAs, robustly present in mitochondria, would be preferable reference genes for expression analysis of mitomiRs.^65^^,^^122^ Zheng et al. used miR-320a to normalize mitomiR expression in mitochondrial extracts as it was abundantly expressed in mitochondrial extracts and did not change during osteogenic differentiation of human mesenchymal stem cells.^126^ MiR-103a was used to normalize mitomiR expression in mitochondrial extracts of colorectal adenomas.^133^ If multiple subcellular fractions are to be compared, it may not be possible to find an appropriate reference gene due to the different RNA composition of subcellular fractions. To overcome this problem, Wang et al. used the global mean normalization method for comparing miRNA expression in multiple subcellular fractions and found that miR-19b levels did not differ between cytosolic and mitochondrial fractions. They consequently used this miRNA to normalize miR-155 and miR-223 expression in hippocampal cytosolic and mitochondrial fractions.^130^

The addition of synthetic spike-in miRNA standards is a good alternative allowing normalization against an external control.^65^^,^^122^ Spike-in miRNAs are added at determined concentrations (within the linear dynamic range of the assay) immediately after lysis of the original sample, and therefore serve as readouts of technical variation throughout the whole experimental procedure, from RNA purification to RT-qPCR or library preparation and RNA-seq. On the contrary, spike-in standards do not allow correction against biological variability between samples, and their combined use with internal reference genes is recommended when comparing independent experiments.

Quantification of miRNA molecules per mitochondria or per cell is more informative on the biological relevance of gene expression changes. This approach involves the generation of standard curves for the miRNA of interest, as well as mitochondrial and nuclear DNA that are used for normalization.^65^ Alternatively, the expression levels of miRNAs per cell or mitochondria can be assessed by quantitative fluorescence imaging (see functional analyses).

#### Bioinformatic analyses

**Figure 3:**
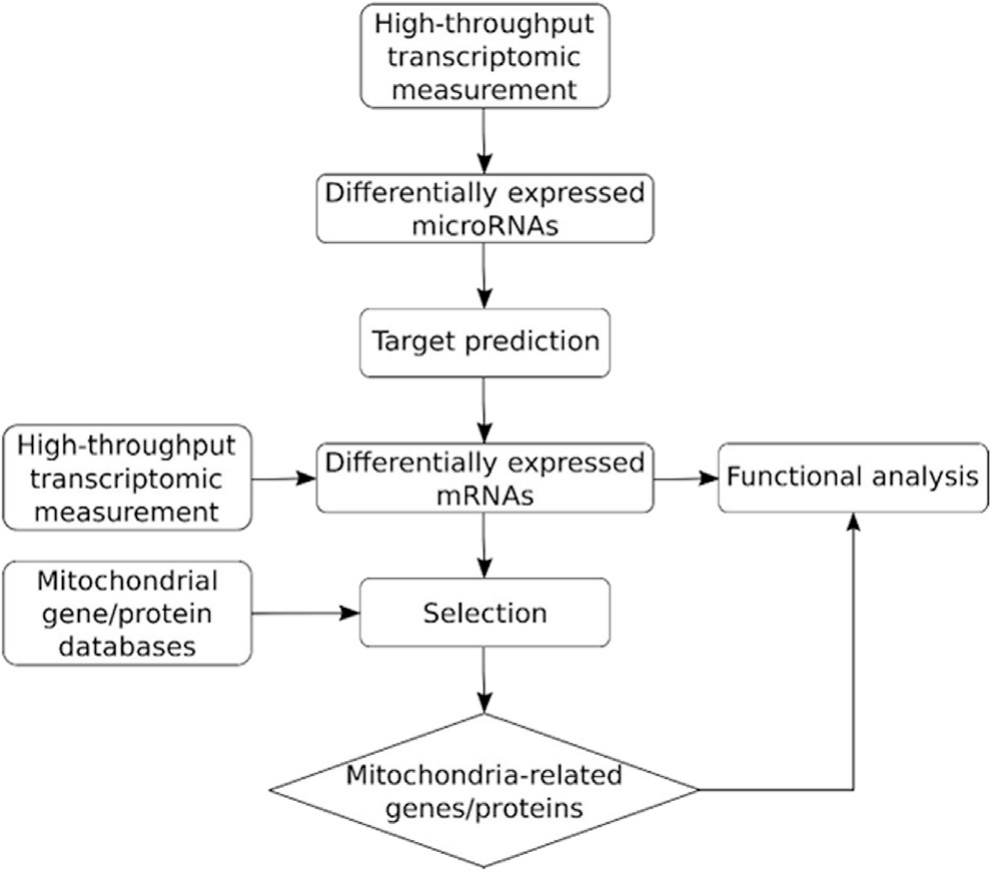
*In silico* analysis of mitochondria-related ncRNAs Possible approaches to retrieve expression profiles of mitochondria-related RNAs from bulk transcriptomic data. Appropriate functional tests will confirm the biological relevance of the differentially expressed miRNAs and validate the link between these miRNAs and their predicted mRNA targets.

Bioinformatic methodologies could be applied to narrow down the bulk transcriptomic data to the expression profile of mitochondria-related RNAs. For this purpose, the most widespread approach is to use mitochondrial gene/protein databases that integrate various sources on mitochondrial involvement.^134^^,^^135^^,^^136^ Mitochondrial gene/protein databases, such as MitoCarta3.0 or MitoMiner4.0, provide information on genes encoding mitochondria-localized proteins or proteins involved in mitochondria-related processes, based on experimental data (e.g., green fluorescent protein tagging and mass spectrometry), manual curation (literature and public databases), and computational evidence (Table 5).^134^^,^^135^^,^^136^^,^^137^^,^^138^^,^^139^ Using such mitochondrial gene/protein databases, differentially expressed genes derived from RNA-seq can be filtered for mitochondria-related differentially expressed genes (Figure 3).^140^^,^^141^^,^^142^^,^^143^

**Table 5:** Mitochondrial gene/protein databases for identifying mitochondria-related genes in bulk transcriptomic datasets

Database name (latest release)	Available species	Evidence category	Reference
MitoCarta3.0 2020	human and mouse	• experimentally validated • manually curated from public databases and literature • computational predictions	Rath et al.^139^
MitoMiner4.0 2018	several species including human, mouse, and rat	• experimentally validated • manually curated from public databases and literature • computational predictions	Smith and Robinson^138^
Integrated Mitochondrial Protein Index (IMPI) 2021	several species including human, mouse, and rat	• MitoMiner4.0 data • machine-learning-based predictions	Smith et al.^144^
MitoProteome 2022	several species including human, mouse, and rat	• experimentally validated • manually curated from public databases and literature	Cotter et al.^145^

Alternatively, non-specific sources (e.g., UniProt, large-scale projects like Human Protein Atlas), pathway (e.g., Reactome, Kyoto Encyclopedia of Genes and Genomes), and functional (e.g., Gene Ontology, MSigDB) databases also provide information on subcellular localization and function of mitochondrial RNA-encoded proteins, and thus can be utilized to examine bulk transcriptomic datasets to identify dysregulated mitochondria-related genes.^134^^,^^146^^,^^147^^,^^148^^,^^149^^,^^150^^,^^151^^,^^152^^,^^153^ These non-specific sources are often integrated in specific mitochondrial gene/protein databases as well.^138^ Gene ontology and pathway enrichment analysis tools can be used to analyze the narrowed-down mitochondria-related gene sets to provide further specific functional information on the genes of interest.^142^^,^^143^^,^^154^^,^^155^

As ncRNAs and especially miRNAs constitute major regulators of gene expression, identification of differentially expressed mitochondria-related genes can be obtained indirectly from miRNA-seq data by predicting mitochondria-related targets for differentially expressed miRNAs.^156^^,^^157^^,^^158^ With genome-wide target prediction using experimentally validated miRNA-target interaction databases (e.g., miRTarBase, miRecords) and/or prediction algorithms (e.g., PicTar, miRanda, DIANA-microT, TargetScan), genes likely regulated by the miRNAs of interest can be retrieved.^159^^,^^160^^,^^161^^,^^162^^,^^163^^,^^164^ Sequence-based prediction tools for miRNA targets rely on complementarity of base pairing and evolutionary conservation. There is no perfect algorithm, especially for identifying targets of novel miRNAs. Combining multiple tools increases specificity of miRNA target recognition at the expense of sensitivity. Selection of the appropriate tool also depends on the mRNA region (UTR, CDS) to be scanned.^165^^,^^166^^,^^167^^,^^168^^,^^169^ As an alternative approach, mitochondrial genome-wide miRNA-target predictions rely on different sequence-based prediction algorithms to scan the mitochondrial genome for possible targets of the investigated miRNAs.^157^^,^^170^^,^^171^

These approaches can be used to define possible actions of mitochondrial RNAs. Although bioinformatic approaches that are capable of determining the localization of RNAs in mitochondria exist, the accuracy and the coverage of these approaches could be further increased.^172^^,^^173^

### Functional analyses

Mitochondria-related ncRNAs have been linked to the regulation of different aspects of mitochondrial physiology, in particular mitochondrial bioenergetics, metabolic reprogramming, and the expression/function of specific electron transport chain (ETC) subunits (summarized in Tables 1 and S1).^4^^,^^42^^,^^46^^,^^174^^,^^175^ Regulation of mitochondrial metabolism by ncRNAs is mediated at multiple levels of gene expression, including transcription, RNA degradation, translation, protein import into mitochondria, and assembly of respiratory complexes.

As previously discussed, the majority of known mitochondria-related miRNAs affect the stability of their mRNA targets, leading to the downregulation of ETC subunits, reduced mitochondrial respiration and increased ROS production.^5^^,^^24^^,^^27^^,^^29^^,^^36^ Other miRNAs, such as miR-1, miR-21, and miR-5787, regulate mitochondrial respiratory chain complexes by promoting the translation of specific subunits.^30^^,^^31^^,^^32^ miR-2392 downregulates mitochondrial genome-encoded ETC subunits by repressing mtDNA transcription.^33^ circRNA mcPGK1 mediates a metabolic switch from oxidative phosphorylation to glycolysis by promoting the import of PGK1 into mitochondria, whereas circPUM1 regulates mitochondrial respiratory chain complex III assembly by directly binding to UQCRC2.^40^^,^^41^ Lastly, some examples of cross-regulation between ncRNAs have been documented. In particular, lncRNAs Kcnq1ot1 and Cerox1 inhibit miR-378a and miR-488-3p, respectively, and consequently upregulate the expression of the mRNA targets of these miRNAs.^36^^,^^37^

In the following paragraphs, we discuss the available systems and methodology to study the impact of ncRNAs on the function of mitochondria (Figure 2F). We emphasize critical points that could explain discrepancies among studies and should thus be considered when addressing the functional role of ncRNAs in mitochondria. We also discuss bioimaging approaches for the detection and quantification of mitochondria-localized ncRNAs and the current methodology for modulating their expression levels.

#### Experimental systems and spatial considerations for functional analyses of RNAs

Both *in vivo* (animal models) and *in vitro* (primary cell cultures, cell lines, differentiated cells from embryonic stem cells, or induced pluripotent stem cells, 2D/3D models) can be used for the study of RNAs in mitochondria (for studies in cardiomyocytes, neurons, and hepatocytes).^176^^,^^177^^,^^178^^,^^179^^,^^180^^,^^181^ Combining information from different models is essential for elucidating the biological function and deciphering the molecular mechanisms by which specific ncRNAs affect mitochondrial physiology.

Mature cells such as high-energy-demanding cardiomyocytes, neurons, and hepatocytes display spatially distinct mitochondrial subpopulations.^182^^,^^183^^,^^184^ In cardiac cells, mitochondria are classified as subsarcolemmal, nuclear, and/or interfibrillar. Subsarcolemmal and nuclear mitochondria lack specific organization. On the contrary, interfibrillar mitochondria are mainly tubular and are highly organized within contractile filaments. In addition to their morphological differences, these subpopulations show distinct metabolic profiles and differential responses to physiological stimuli, such as apoptotic signals.^185^^,^^186^^,^^187^^,^^188^ As expected, interfibrillar mitochondria display high oxidative phosphorylation activity to provide the necessary energy for contraction. These features suggest that ncRNAs may have differential effects on cardiac mitochondrial subpopulations and underline the importance of using specific protocols to isolate and analyze separately these subpopulations.^188^^,^^189^ Such an approach demonstrated that a pool of mitomiRs, including miR-378 that targets the ATP synthase subunit ATP6, shows differential redistribution between subsarcolemmal and interfibrillar cardiac mitochondria in response to diabetes.^5^^,^^30^

A novel approach, permeabilized cell mitochondrial function sequencing (PMF-seq), was recently developed to study the genetic profile of distinct mitochondrial subpopulations.^190^ In PMF-seq, cells are subjected to gentle permeabilization, which preserves mitochondrial physiology, selected for desired bioenergetic parameters using flow cytometry, and analyzed by NGS. This approach was successfully used to screen for genes that regulate mitochondrial respiration in a pool of CRISPR-mutagenized cells, but could also be combined with mitochondrial ncRNA profiling in the future.

#### Bioimaging of mitochondria and mitochondrial ncRNAs

Several imaging strategies for RNAs have been developed. One of them is single-molecule fluorescence *in situ* hybridization (smFISH), which gives information on RNA localization and quantification within the cell. The disadvantages of this method are linked to fluorophore limitations and rapid bleaching of the signal by lasers as well as to the necessary fixation (live imaging is not possible). When combined with immunostaining strategies, smFISH can be used to colocalize fluorescently labeled RNA into a specific cell compartment.^191^ This approach allows monitoring the localization of both exogenous and endogenous RNA. The smFISH technique was used to build the Multiplexed Error-Robust Fluorescence *in situ* Hybridization tool (Merscope). This novel platform works in high-throughput mode with high spatial resolution and sensitivity and its multiplexing is enhanced due to combinatorial labeling.^192^

Hybridization chain reaction has been lately connected with the photoacoustic imaging technique to detect miR-21, for instance.^193^ The advantage of this approach is its specificity to miR-21 (down to 1 base pair mutation) with a detection limit of 148 pM. Moreover, hybridization chain reaction combined with mass spectrometry was able to detect miR-21 at femto-level concentration (via accumulation of ultrasmall up-conversion nanoparticles).^194^

Another interesting platform that can be used for high-throughput imaging of mitochondrial RNAs in single living cells is Multiplexed Organelles Portrait Barcodes. In this system, core-shell mesoporous silica nanoparticles are biofunctionalized with the organelle-targeting peptide. Linking nanoparticles to dyes such as Cy3, Cy5, or AMCA at different ratios and combining them with molecular beacon detection probes allowed to create a tool that recognized mitomiR-155, -146a, -210, and -34a by confocal laser scanning microscopy. This approach was also successfully tested for miRNA alteration recognition in mitochondria and the endoplasmic reticulum related to Ca^2+^ homeostasis modulation. An advantage of this method is the ability to track real-time dynamic changes that can be applied in pathophysiological research.^195^

#### Assessing the impact of ncRNAs on mitochondria bioenergetics

Mitochondria bioenergetics is primarily analyzed using *in vitro* models, namely intact cells, permeabilized cells, whole-cell homogenates, or mitochondria-enriched fractions. The size of available biological material (tissue biopsy, isolated cells, mitochondrial fractions) is critical for the design of research protocols. The use of permeabilized cells combines several advantages: all mitochondria subpopulations are taken into account, mitochondrial networks and their interactions with other organelles are maintained, and, as opposed to the use of intact cells, the experimenter has direct control on the medium surrounding mitochondria.^196^ A titration assay of the permeabilization reagent (saponin) is required to avoid outer mitochondrial membrane damage (evidenced by cytochrome *c* release), taking into account that the assay buffer used for the subsequent analysis may have an additive effect on the degree of permeabilization.^197^ On the other hand, the use of mitochondrial fractions allows identifying defects on specific components of the ETC, which can be further confirmed and characterized by specialized enzymatic and assembly assays of mitochondrial respiratory complexes.^198^^,^^199^

Mitochondrial respiration can be analyzed by a variety of respirometry methods that measure oxygen consumption rate as a readout of oxidative phosphorylation.^200^^,^^201^^,^^202^^,^^203^^,^^204^ Seahorse analyzers are adapted for small samples, such as human biopsies. They allow rapid metabolic profiling of cells that goes beyond oxidative phosphorylation (glycolysis, fatty acid oxidation, glutaminolysis) and are ideal for high-throughput screens. Their use is limited by their high cost and the small number of possible injections (up to four). Clark-type electrodes, on the other hand, are inexpensive tools that allow multiple injections for a more precise characterization of the mitochondrial respiration phenotype. They are not suitable for small samples or for comparing multiple conditions simultaneously. The procedure is quite long, and the quality of the sample may decline during the analysis. Oxygraph 2k respirometry shares the same advantages with Clark-type electrode respirometry, with the addition of high sensitivity and precision, and minimal noise. The user benefits from great flexibility to adapt the protocol to specific questions and can combine respirometry with fluorometry to measure Ca^2+^ buffering and mitochondrial membrane potential. Recently, respirometry protocols have been optimized and adapted for the use of thawed samples to overcome the challenges of immediate sample processing and bioenergetics analysis in the clinical setting.^205^^,^^206^

Overall, these methods can provide insight into different aspects of how RNAs regulate mitochondrial bioenergetics. Their combined use can give complementary information. In addition to the nature of the sample used, the composition of the assay buffer can influence the analysis. Data correction is an important parameter to consider to avoid discrepancies and, when possible, normalization against mitochondrial content (mtDNA, citrate synthase activity) should be applied instead of normalization against total protein or tissue mass.^206^

Finally, other aspects of mitochondrial physiology that are tightly linked to mitochondrial respiration, such as mitochondrial Ca^2+^ import, mitochondrial ROS production, and mitochondrial membrane potential, should be analyzed to get a better insight into the regulation by RNAs of the observed phenotypes.^207^^,^^208^ For example, overexpression of miR-181c led to the reduction of its target, mt-COX1, as well as other mitochondrial respiratory complex IV subunits, and to complex IV remodeling. Surprisingly, the effect on mitochondrial respiration was the opposite of what would have been expected, as it was enhanced, due to a parallel increase in mitochondrial Ca^2+^ import.^24^

Characterization of mitochondrial bioenergetics *in vivo* is possible using non-invasive, magnetic resonance spectroscopy (MRS)-based methods.^209^ ^31^P-MRS allows measuring the phosphocreatine versus ATP (PCr/ATP) ratio, which is a readout of the energetic state of the heart. When combined with ^13^C-MRS it detects oxidation products and allows the evaluation of mitochondria coupling of fatty acid oxidation with ATP synthesis. These methods are far from being widely applied as they are time-consuming and lack resolution and mechanistic insight.

#### Mitochondria-targeted RNA therapies and delivery strategies

As mentioned previously, mitochondrial ncRNAs play crucial roles in controlling various disease processes by modulating glycolysis, mitochondrial respiration, and the expression of genes involved in mitochondrial metabolism and homeostasis. Standardizing methods for targeting/modulating mitochondrial ncRNAs has great potential in enhancing our understanding of the underlying mechanisms linking fluctuations in their expression levels to disrupted mitochondrial function and specific disease phenotypes, as well as in promoting the development of mitochondria-targeted RNA therapies.

RNA-based therapies have shown remarkable efficacy in treating certain well-defined genetic conditions, and include RNA interference, aptamers, antisense oligonucleotides (ASOs), small interfering RNAs (siRNAs), CRISPR-based gene editing, and mRNA therapeutics.^210^^,^^211^ The number of FDA-approved RNA drugs has seen a rapid increase over the past decade (Table S2); also, hundreds of novel investigational drugs are in the preclinical stage or already progressing in the advanced phase of clinical trials. Although RNA-based therapies for mitochondrial diseases have yet to receive approval, targeting mitochondrial RNAs holds promise for therapeutic interventions, by leveraging (1) ASOs and siRNAs to decrease mutant mitochondrial protein levels, (2) mRNA-based drugs to substitute defective mitochondrial RNAs and proteins, and (3) anti-replicative RNAs to mitigate mutant mtDNA levels in cells.^212^ Delivery of siRNAs, mitomiR mimics, or ASOs to mitochondria can modulate mitochondrial gene expression for specific therapeutic purposes. For instance, nanocarriers have been utilized to transport let-7b to mitochondria, reducing the expression of COX1 and COX2 in non-small cell lung cancer cells.^213^ ASOs have also been transported to the mitochondrial matrix using MITO-porter, effectively decreasing COX2 mRNA expression.^214^

In addition, gene editing approaches offer the potential for treating mitochondrial diseases. Various tools have been developed for modifying mtDNA, including bacterial toxin derivatives such as DddA-derived cytosine base editor, which can introduce single-base changes.^215^ Protein-only nucleases, such as mitoTALEN (transcription activator-like effector nuclease), equipped with mitochondrial targeting sequences, can be imported into the mitochondrial matrix for specific mtDNA editing.^216^ In addition, CRISPR-Cas9 systems have been adapted for targeting mtDNA (mitoCRISPR).^217^ While there is growing evidence of evolving mitochondrial therapeutic delivery systems, RNA import into mitochondria remains largely understudied. Further research is necessary to elucidate the fundamental principles underlying RNA import into mitochondria.

## Conclusion

Mitochondria are organelles of utmost importance in all cells, particularly in high-energy-demanding cells. The cellular transcriptome is much more complex than previously anticipated, with many RNAs having regulatory properties. ncRNAs unable to encode proteins regulate gene expression at multiple stages and thereby participate in virtually all pathophysiological processes. A detailed knowledge of the role of RNAs may lead to the discovery of novel therapeutic targets. Circulating RNAs also have shown some biomarker potential in many diseases. Mitochondria possess a pool of protein-coding and ncRNAs with poorly understood functions. This partial and sometimes debated knowledge arising from inconsistent studies may come from the use of diverse protocols for mitochondria purification, RNA extraction and quantification, and functional assessments. By standardizing these methods, we can certainly reduce findings inconsistency, improve the state-of-the-art of the role of mitochondrial RNAs in disease development and progression, and generate reliable and robust research findings that can be translated to clinical application for the benefit of patients. Fulfilment of technical and experimental guidelines has the potential to move personalized medicine a step forward. After the use of RNAs for vaccine and diagnostic tests during the COVID-19 pandemic, RNAs may well become the next generation of drugs and biomarkers.

## Supplemental information

Document S1. Tables S1 and S2

Document S2. Article plus supplemental information
