# Supplementary material for: Guidelines for mitochondrial RNA analysis
Source: Mol Ther Nucleic Acids. 2024 Jun 26;35(3):102262. doi: 10.1016/j.omtn.2024.102262 (PMC11292373; doi:10.1016/j.omtn.2024.102262)
Supplement: Document S1. Tables S1 and S2 [file mmc1.pdf]

OMTN, Volume *35*

Supplemental information

Guidelines for mitochondrial RNA analysis

Amela Jusic, Zoi Erpapazoglou, Louise Torp Dalgaard, Päivi Lakkisto, David de Gonzalo-Calvo, Bettina Benczik, Bence Ágg, Péter Ferdinandy, Katarzyna Fiedorowicz, Blanche Schroen, Antigone Lazou, Yvan Devaux, on behalf of EU-CardioRNA COST Action CA17129, and AtheroNET COST Action CA21153

Table S1. Summary of miRNAs targeting mitochondrial genes.

miRNA	Origin	Mitochondrial gene	Reference
hsa-miR-mit3, hsa-miR-mit6, and hsa-miR-mit4	Mitochondria	MT-RNR1 (12S rRNA) and MT-RNR2 (16S rRNA)	[1]
hsa-miR-mit3, hsa-miR-mit3, and hsa-miR-mit4	Mitochondria	MT-ATP6<br>MT-CO3	[1]
mitomiR-2392	Mitochondria	MT-ND2, MT-ND4, MT-ND5, MT-CYB and MT-CO1	[2]
hsa-miR-338	Nucleus	COXIV and ATP5G1	[3]
hsa-miR-146a	Nucleus	16S rRNA, MT-ND1, MT-ND2, MT-ND4, MT-ND5, MT-ND6 and MT-CYB	[4]
hsa-miR-26b, hsa-miR-100, and hsa-miR-143	Nucleus	MT-CO2	[5]
hsa-miR-181c	Nucleus	MT-CO1	[6]

Table S2. FDA-approved RNA-based drugs.

Therapeutic area	Company	Candidate	Modality	Target	Indication
Cardiovascular	Alnylam + Novartis	Leqvio (Inclisiran)	RNAi	Proprotein convertase subtilisin/kexin type 9	Atherosclerosis
	Alnylam + Roche	Zelebesiran	RNAi	Angiotensinogen	Hypertension
Renal	Alnylam	Oxlumo (Lumasiran)	RNAi	Glycolate oxidase	Primary hyperoxaluria type I
Hepatology	Alnylam	Gocsiran	RNAi	5'-amino-levulinate synthase 1	Acute hepatic porphyria
Neuromuscular	NS Pharma	Viltepso (Vitolarsen)	ASO	Dystrophin exon 53	Duchene muscular dystrophy

	Serepta	Exondys 51(eteplirsen)	PMO	Dystrophin exon 51	Duchene muscular dystrophy
Neurology	Alnylam<br>Ionis + PTC	Onpattro (patirsiran) & Amvuttra (vutrisiran)<br>Tegesedi (inotersen)	RNAi<br>ASO	Transthyretin	hATTR Amyloidosis-PN
	Biogen + Ionis	Qaisody (tofersen)	ASO	Superoxide dismutase type 1	SOD-1 Amyotrophic Lateral Sclerosis
Infectious diseases	Moderna	Spikevax (mRNA-1273)	mRNA		Covid-19 vaccine
	Pfizer +BioNTech	Comirnaty	mRNA		Covid-19 vaccine

References

1. Shinde, S., and Bhadra, U. (2015). A Complex Genome-MicroRNA Interplay in Human Mitochondria. BioMed Research International *2015*, 1–13.
2. Fan, S., Tian, T., Chen, W., Lv, X., Lei, X., Zhang, H., Sun, S., Cai, L., Pan, G., He, L., et al. (2019). Mitochondrial miRNA Determines Chemoresistance by Reprogramming Metabolism and Regulating Mitochondrial Transcription. Cancer Research *79*, 1069–1084.
3. Bandiera, S., Matégot, R., Girard, M., Demongeot, J., and Henrion-Caude, A. (2013). MitomiRs delineating the intracellular localization of microRNAs at mitochondria. Free Radic Biol Med *64*, 12–19.
4. Dasgupta, N., Peng, Y., Tan, Z., Ciraolo, G., Wang, D., and Li, R. (2015). miRNAs in mtDNA-less cell mitochondria. Cell Death Discovery *1*, 15004.
5. Ferraz, R.S., Santos, L.C.B., da-Silva-Cruz, R.L., Braga-da-Silva, C.H., Magalhães, L., Ribeiro-Dos-Santos, A., Vidal, A., Vinasco-Sandoval, T., Reis-das-Mercês, L., Sena-Dos-Santos, C., et al. (2022). Global miRNA expression reveals novel nuclear

and mitochondrial interactions in Type 1 diabetes mellitus. Front Endocrinol *13*, 1033809.

6. Das, S., Ferlito, M., Kent, O.A., Fox-Talbot, K., Wang, R., Liu, D., Raghavachari, N., Yang, Y., Wheelan, S.J., Murphy, E., et al. (2012). Nuclear miRNA Regulates the Mitochondrial Genome in the Heart. Circ Res *110*, 1596–1603.
